# Post-COVID recovery is faster after an infection with the SARS-CoV-2 Omicron variant: a population-based cohort study

**DOI:** 10.1007/s15010-024-02438-z

**Published:** 2024-11-18

**Authors:** Laura Rebecca Pfrommer, Sophie Diexer, Bianca Klee, Janka Massag, Cornelia Gottschick, Oliver Purschke, Mascha Binder, Thomas Frese, Matthias Girndt, Daniel Sedding, Jonas Rosendahl, Jessica I. Hoell, Irene Moor, Michael Gekle, Christine Allwang, Florian Junne, Rafael Mikolajczyk

**Affiliations:** 1 Institute for Medical Epidemiology, Biometry and Informatics (IMEBI), Interdisciplinary Centre for Health Sciences, Medical Faculty of the Martin Luther University Halle-Wittenberg, Halle (Saale), Germany; 2 Division of Medical Oncology, University Hospital Basel, Basel, Switzerland; 3 Institute of General Practice and Family Medicine, Interdisciplinary Centre for Health Sciences, Medical Faculty of the Martin Luther University Halle-Wittenberg, Halle (Saale), Germany; 4 Department of Internal Medicine II, Martin Luther University Halle-Wittenberg, Halle (Saale), Germany; 5 Mid-German Heart Centre, Department of Cardiology and Intensive Care Medicine, University Hospital, Martin Luther University Halle-Wittenberg, Halle (Saale), Germany; 6 Department of Internal Medicine I, Martin Luther University Halle-Wittenberg, Halle (Saale), Germany; 7 Paediatric Haematology and Oncology, Martin Luther University Halle-Wittenberg, Halle (Saale), Germany; 8 Institute for Medical Sociology, Interdisciplinary Centre for Health Sciences, Medical Faculty of the Martin Luther University Halle-Wittenberg, Halle (Saale), Germany; 9 Julius-Bernstein-Institute of Physiology, Medical Faculty of the Martin Luther University Halle-Wittenberg, Halle (Saale), Germany; 10 Department of Psychosomatic Medicine and Psychotherapy, Klinikum rechts der Isar, Technical University Munich, Munich, Germany; 11 Department of Psychosomatic Medicine and Psychotherapy, Otto-von-Guericke-University Magdeburg, Magdeburg, Germany

**Keywords:** Post Acute COVID-19 syndrome, Long Haul COVID-19, SARS-CoV-2 virus variants, COVID-19 vaccine, COVID-19 recovery

## Abstract

**Purpose:**

Post-COVID-19 condition (PCC) poses a substantial burden to affected individuals, health care systems, and society as a whole. We examined factors associated with recovery from PCC, focusing on the vaccination status prior to infection and the virus variant.

**Methods:**

Our analyses are based on the population-based cohort study for digital health research in Germany (DigiHero). Respondents who reported a SARS-CoV-2 infection and COVID-related symptoms ≥ 12 weeks post-infection were classified as having PCC. Those with ongoing PCC were followed-up in six-month intervals based on their date of infection. We used a Cox model for interval-censored data to analyze PCC recovery.

**Results:**

Among the 4,529 respondents with PCC included in our analyses, about 26%, 19%, 36%, and 44% of those infected during dominance of the SARS-CoV-2 wildtype, Alpha, Delta, and Omicron variant had recovered one year after infection, respectively. When stratifying by virus variant, vaccination was not associated with a faster recovery. Conversely, those infected with Omicron (HR = 2.20; 95%CI: 1.96–2.48) or Delta (HR = 1.69; 95%CI: 1.43–2.01) recovered faster than those infected with the SARS-CoV-2 wildtype or Alpha strain.

**Conclusion:**

Although the recovery from PCC is faster for the newer virus variants, still a substantial fraction of those who developed PCC after an infection with the Omicron variant report prolonged persistence of symptoms.

**Supplementary Information:**

The online version contains supplementary material available at 10.1007/s15010-024-02438-z.

## Introduction

It was recently estimated that nearly 30% of COVID-19 survivors still experience long-lasting symptoms even two years after SARS-CoV-2 infection [1]. Post-COVID-19 condition (PCC) refers to symptoms that persist at least 12 weeks after acute infection for at least two months and cannot be explained by other conditions [2]. Those affected report a variety of symptoms, that profoundly impact their quality of life and their ability to perform daily activities and to participate in the workforce [3–5]. While several studies have addressed risk factors for PCC [3, 6–17], research on factors associated with PCC recovery is still limited.

Previous studies suggested a lower risk of developing PCC for infection with the Omicron variant compared to earlier variants [3, 6, 8, 11–13, 16]. However, it is not clear whether this also means that recovery for those with PCC is faster for infections caused by Omicron and what role vaccination status plays in PCC recovery. As for the risk of developing PCC, a protective effect of SARS-CoV-2 vaccination is assumed [17, 18]. However, some recent research did not confirm this [6–10, 13, 15]. A potential indirect pathway between vaccination status and PCC risk is currently discussed, suggesting that the vaccination’s ability to mitigate COVID-19 severity may indirectly affect PCC risk [3, 12].

We propose that this mechanism extends to PCC recovery, and that observed associations between vaccination and recovery may mainly result from the prevailing virus variant, given the likely high correlation between these two factors. Thus, we investigated factors associated with PCC recovery, focusing on virus variants and vaccination status.

## Methods

### The post-COVID subcohort of the DigiHero study

We used data from the German population-based prospective cohort study for digital health research (DigiHero). DigiHero has been described elsewhere [19]. In brief, participants randomly selected for study participation were invited by regular mail; the subsequent study participation was digital. Socio-demographic aspects were assessed at baseline (rollout January 2021). We followed up on SARS-CoV-2 infections and complaints at ≥ 12 weeks post-infection (infections-assessment, rollout August 2021). Here, we presented a list of 24 complaints (Online Resource Table 1) and asked how severe those were (“don’t know“, “very mild”, “mild”, “moderate”, “severe”). Respondents should further specify the perceived general course of their acute disease (“no symptoms”, “mild”, “moderate”, “severe”). We also asked if they currently had ongoing symptoms. We initiated a PCC-registry to follow-up participants with ongoing PCC in six-monthly time intervals based on their date of infection. In this analysis, we use data up to the first follow-up within the registry (follow-up assessment, rollout December 2022).

#### Post-COVID definition

Those with any of the 24 self-reported COVID-related symptoms ≥ 12 weeks post-infection, regardless of the reported degree of severity, were considered PCC cases and form the basis for our analyses. We considered those who reported not experiencing ongoing symptoms at the infections-assessment or the follow-up as “PCC recovered” within the respective time interval.

### Data preparation

We included respondents who completed the baseline assessment by May 25th, 2023 and considered information on the follow-up until March 4th, 2024. We excluded respondents with missing information on age, sex, date of infection, vaccination status, or ongoing symptoms at the infections-assessment, as well as respondents who reported “diverse” sex and those with implausible age, infection date (in the future or before January 28th, 2020), or vaccination date (before vaccine roll-out, distance between two doses less than two weeks).

We considered the reported date of the first positive test as an estimate for the respective date of infection. When multiple infections were reported, we considered the infection after which PCC was reported for the first time. We further determined the vaccination status at time of infection (not vaccinated, one dose, two doses, three or more doses).

We estimated the virus variant based on the dominant variant of concern at the time of infection in Germany [20].

### Statistical analysis

We report relative and absolute frequencies for categorical, and median and interquartile range (IQR) for metric variables. As the data is interval-censored (recovery was possible between 12 weeks post-infection and the infections-assessment or between the infections-assessment and follow-up), we used the non-parametric maximum likelihood estimate and conducted Cox regression analysis for interval-censored data using the R package *icfit* [21]. First, we conducted a crude analysis only considering vaccination status and then adjusted for sex, age, educational level, and net household income. We then stratified for virus variant. Finally, we repeated Cox regression considering virus variant instead of vaccination status. Variables included in the models were assessed for multicollinearity.

#### Sensitivity analyses

Since the course of acute infection is reported to be an important risk factor for PCC [7–10, 16, 17, 22], we repeated survival analysis, including this variable. This was only possible for a subpopulation (Baseline completion by July 2022). Furthermore, we repeated our analyses only considering those who classified at least one PCC-symptom as “severe”.

## Results

**Fig. 1:**
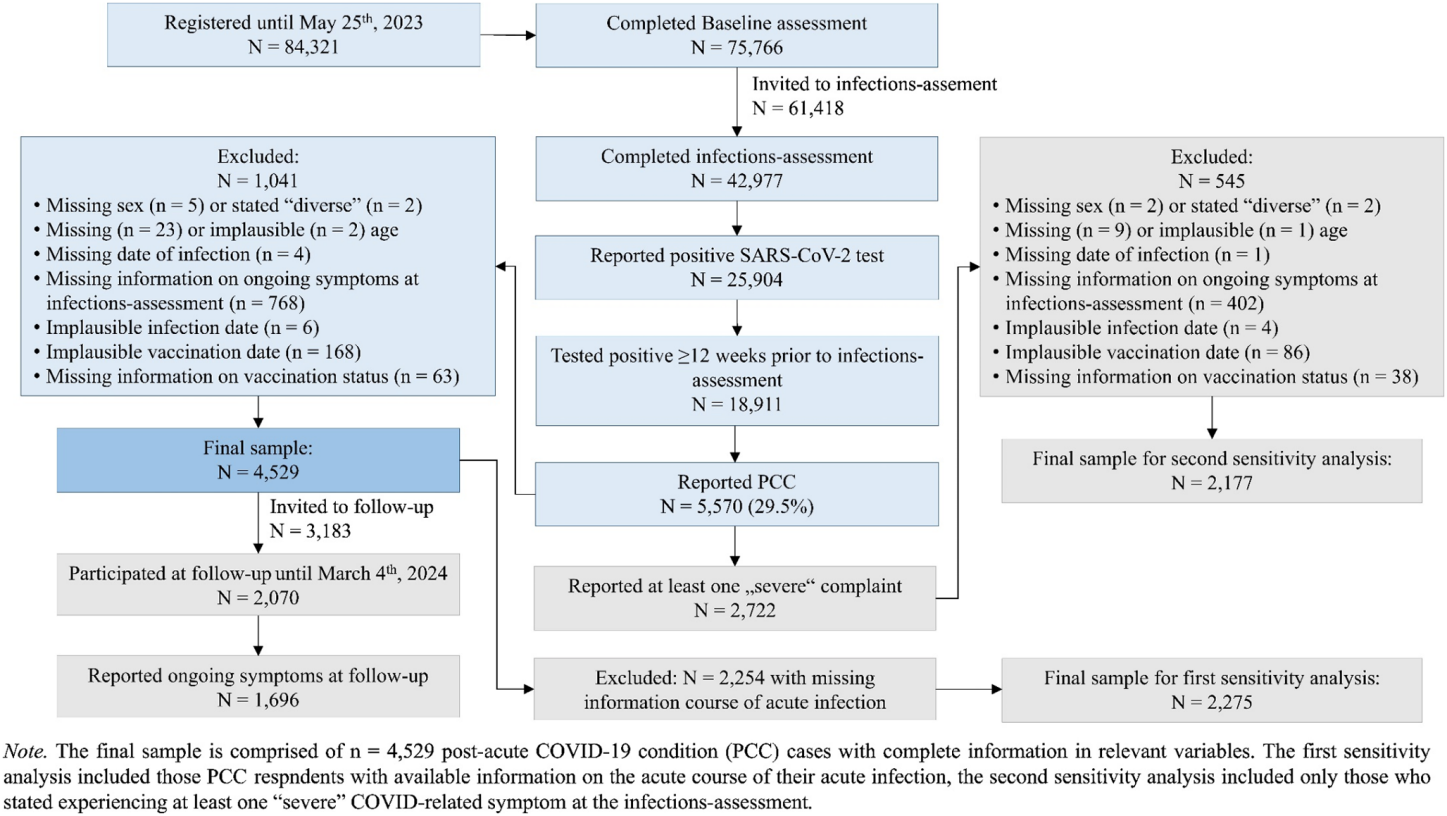
Flow-chart of the study population

Our analyses are based on 4,529 DigiHero participants who were classified as PCC cases (Fig. 1). Their median age was 50 years (IQR = 20) ranging from 18 to 86, 72.6% were women (Table 1). Of all those who reported PCC, 48.9% reported experiencing at least one PCC-symptom they described as “severe”.

**Table 1:** Characteristics of DigiHero respondents with self-reported post-acute COVID-19 condition (any COVID-related symptoms ≥ 12 weeks post-infection)

Respondent characteristics		Included in analyses (*N* = 4,529)		Excluded (*N* = 1,041)	
		n	*%*	n	*%*
Sex	male	1,239	*27.4*	323	*31.0*
	female	3,290	*72.6*	710	*68.2*
	diverse	-	*-*	3	*0.3*
	not available	-	*-*	5	*0.5*
Age	< 30	499	*11.0*	130	*12.5*
	30–39	737	*16.3*	191	*18.3*
	40–49	956	*21.1*	255	*24.5*
	50–59	1,328	*29.3*	276	*26.5*
	60–69	763	*16.8*	127	*12.2*
	≥ 70	246	*5.4*	37	*3.6*
	not available/ implausible	-	*-*	25	*2.4*
Dominant virus variant at time of infection	SARS-CoV-2 wildtype	709	*15.7*	273	*26.2*
	Alpha	651	*14.4*	206	*19.8*
	Delta	605	*13.4*	144	*13.8*
	Omicron	2,564	*56.6*	404	*38.8*
	not available	-	*-*	14	*1.3*
COVID-19 vaccination status prior to infection	not vaccinated	1,593	*35.2*	410	*39.4*
	one dose	136	*3.0*	33	*3.2*
	two doses	692	*15.3*	105	*10.1*
	three or more doses	2,108	*46.5*	250	*24.0*
	not available/ implausible	-	*-*	243	*23.3*
Education level^a^	low	155	*3.4*	43	*4.1*
	medium	1,738	*38.4*	419	*40.2*
	high	2,555	*56.4*	557	*53.5*
	not available	81	*1.8*	22	*2.1*
Net household income in €	< 2.250	918	*20.3*	219	*21.0*
	2.250 to < 4.000	1,680	*37.1*	365	*35.1*
	≥ 4.000	1,519	*33.5*	345	*33.1*
	not available	412	*9.1*	112	*10.8*
Course of acute infection	no symptoms/ mild course	746	*16.5*	149	*14.3*
	moderate course	1,144	*25.3*	252	*24.2*
	severe course	385	*8.5*	94	*9.0*
	not available	2,254	*49.8*	546	*52.5*
Information on self-reported Post-COVID-19 condition	recovered between 12 weeks and IA	1,346	*29.7*	65	*6.2*
	recovered between IA and FU	374	*8.3*	70	*6.7*
	right censored at FU	1,696	*37.4*	287	*27.6*
	right censored at IA	1,113	*24.6*	83	*8.0*
	not available	-	*-*	536	*51.5*

Note. Respondents could either recover between 12 weeks post-infection and the infections-assessment (IA) or between the infections-assessment and the follow-up (FU), with the rollout of the FU being dependent on the individual infection date

^a^ The education level was defined based on the International Standard Classification of Education (ISCED-97) [23]

### Post-COVID recovery

**Fig. 2:**
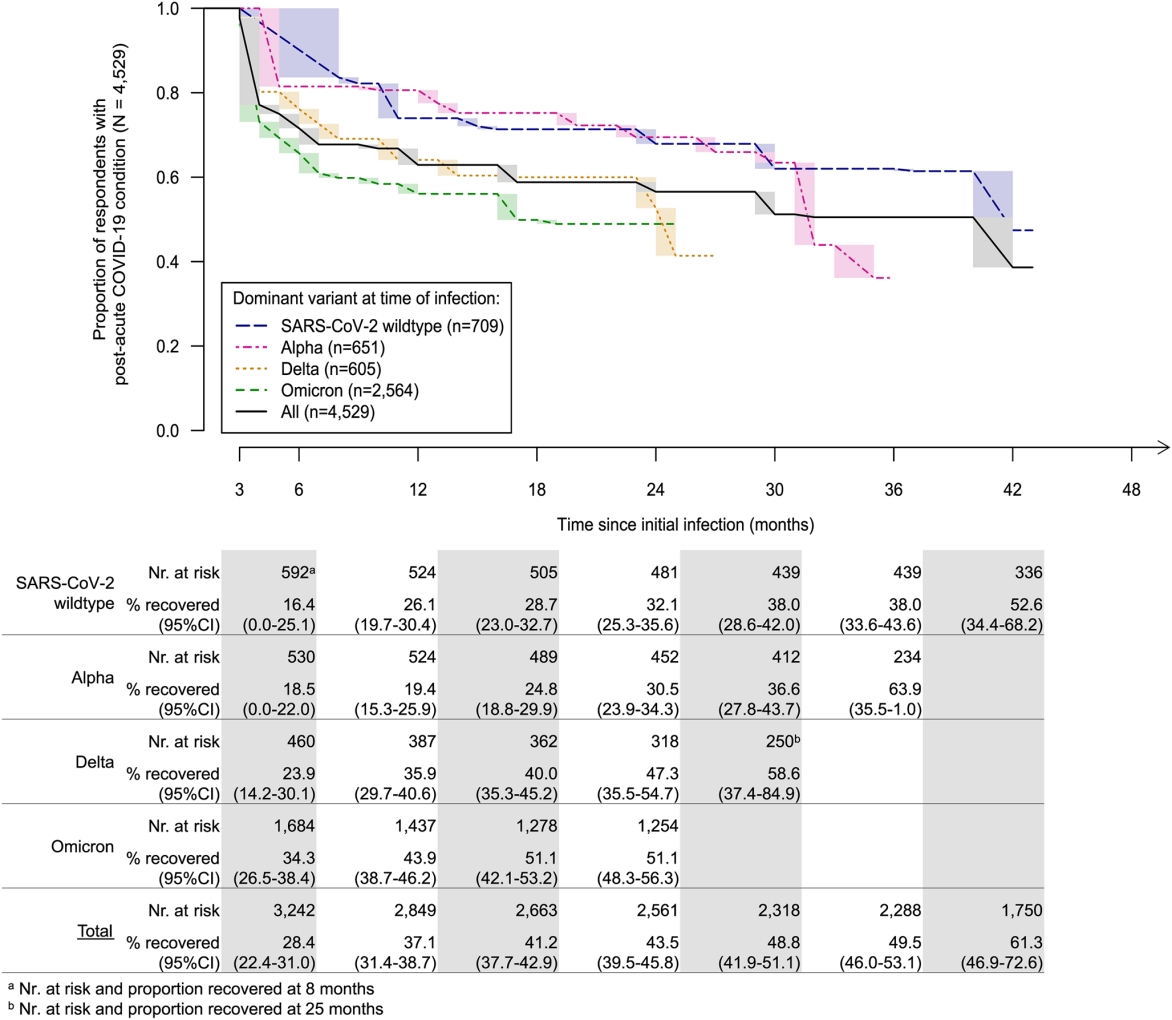
Recovery from post-acute COVID-19 condition in the DigiHero cohort (non-parametric maximum likelihood estimates for dominant SARS-CoV-2 variant at time of infection. Each potential curve within the shaded areas is equally likely)

While for the SARS-CoV-2 wildtype and the Alpha variant recovery was similar, recovery was faster for the newer variants (Fig. 2). Thus, one year after infection, 26.1% (95% confidence interval [95%CI]: 19.7–30.4) of those infected with the SARS-CoV-2 wildtype were recovered, while this was the case for 19.4% (95%CI: 15.3–25.9) of those infected with the Alpha, 35.9% (95%CI: 29.7–40.6) of those infected with the Delta, and 43.9% (95%CI: 38.7–46.2) of those infected with the Omicron variant. Of all PCC respondents, 37.1% (95%CI: 31.4–38.7) recovered within a year, 43.5% (95%CI: 39.5–45.8) within two years after infection. As issues with the proportional hazards assumption became evident when depicting the nonparametric maximum likelihood estimates (Fig. 2), we combined infection with the SARS-CoV-2 wildtype or Alpha for the multivariable analysis.

When considering COVID-19 vaccination status, while not accounting for the virus variant (Table 2, Panel A), having received two or more vaccine doses was associated with a faster recovery than not being vaccinated (e.g., hazard ratio [HR]_two doses vs. not being vaccinated_ = 1.50; 95%CI: 1.31, 1.72). However, when repeating this analysis stratified by virus variant, this effect disappeared (Table 2, Panel B). We then repeated Cox regression analysis, considering virus variant instead of vaccination status (Table 2, Panel C). Compared to an infection with the SARS-CoV-2 wildtype or Alpha variant, a faster PCC recovery was present for those infected with Delta (HR = 1.69; 95%CI: 1.43, 2.01) or Omicron (HR = 2.20; 95%CI: 1.96, 2.48).

**Table 2:** Factors associated with time to recovery from post-acute COVID-19 condition (Cox regression for interval-censored data; numbers above 1 indicate a faster recovery)

**A. Considering vaccination status, not variant (** ***N*** ** = 4,529)**	**crude HR**	**95%CI**		**adjusted HR** ^a^	**95%CI**	
Vaccination status prior to infection (ref: not vaccinated, *n* = 1,593)						
Received one dose (*n* = 136)	1.09	0.77	1.55	1.10	0.78	1.56
Received two doses (*n* = 692)	1.51	1.31	1.73	1.50	1.31	1.72
Received booster dose (*n* = 2,108)	1.94	1.73	2.17	1.89	1.69	2.12
**B. Analyses stratified for virus variant**	**crude HR**	**95%CI**		**adjusted HR** ^a^	**95%CI**	
**B.1 Omicron variant only (** ***N*** ** = 2, 564)**						
Vaccination status prior to infection (ref: not vaccinated, *n* = 114)						
Received one dose (*n* = 48)	0.59	0.31	1.09	0.56	0.30	1.04
Received two doses (*n* = 322)	0.87	0.63	1.22	0.82	0.58	1.14
Received booster dose (*n* = 2,080)	0.99	0.74	1.32	0.94	0.70	1.27
**B.2 Delta variant only (** ***N*** ** = 605)**						
Vaccination status prior to infection (ref: not vaccinated, *n* = 175)						
Received one dose (*n* = 43)	0.98	0.56	1.74	0.92	0.51	1.66
Received two doses (*n* = 359)	0.93	0.68	1.28	0.92	0.66	1.29
Received booster dose (*n* = 28)	0.66	0.27	1.61	0.61	0.25	1.48
**B.3 Alpha variant only (** ***N*** ** = 651)**						
Vaccination status prior to infection (ref: not vaccinated, *n* = 595)						
Received at least one dose (*n* = 56)	0.80	0.43	1.47	0.85	0.46	1.58
**B.4 SARS-CoV-2 wildtype or Alpha variant (** ***N*** ** = 1, 360)**						
Vaccination status prior to infection (ref: not vaccinated, *n* = 1,304)						
Received at least one dose (*n* = 56)	0.77	0.41	1.43	0.84	0.44	1.57
**C. Considering variant, not vaccination status (** ***N*** ** = 4,529)**	**crude HR**	**95%CI**		**adjusted HR**	**95%CI**	
**Dominant virus variant at time of infection** (ref: SARS-Cov-2 wildtype or Alpha, *n* = 1,360)						
Delta (*n* = 605)	1.63	1.37	1.94	1.69	1.43	2.01
Omicron (*n* = 2,564)	2.24	1.99	2.52	2.20	1.96	2.48
**Sex** (ref: male; *n* = 1,239)						
Female (*n* = 3,290)				0.81	0.73	0.90
**Age** (ref: <30; *n* = 499)						
30–39 (*n* = 737)				0.79	0.66	0.94
40–49 (*n* = 956)				0.59	0.48	0.72
50–59 (*n* = 1,328)				0.60	0.50	0.72
60–69 (*n* = 763)				0.64	0.52	0.78
≥ 70 (*n* = 246)				0.62	0.46	0.82
**Education level** (ref: high; *n* = 2,555) ^b^						
Low (*n* = 155)				0.99	0.74	1.34
Medium (*n* = 1,738)				0.91	0.82	1.01
Not available (*n* = 81)				0.72	0.46	1.13
**Net household income in €** (ref: <2.250; *n* = 918)						
2.250 to < 4.000 (*n* = 1,680)				1.06	0.93	1.21
≥ 4.000 (*n* = 1,519)				1.24	1.08	1.42
Not available (*n* = 412)				1.05	0.87	1.26

Note. Crude Hazard ratios (HR) as well as adjusted HR and respective 95% confidence intervals (95%CI) are shown. Panel C also depicts the HR for each variable included in the multivariable analysis, while in Panel A and B the HR for the variables sex, age, education level, and household income are not shown

^a^ Analyses adjusted for age, sex, education level, and household income

^b^ The education level was defined based on the International Standard Classification of Education (ISCED-97) [23]

These differences across variants remained apparent in the sensitivity analyses (Online Resource Tables 2 AND 3). Time to recovery was substantially longer for those with a moderate (HR = 0.68; 95%CI: 0.59, 0.79) or severe acute course of infection (HR = 0.33; 95%CI: 0.24, 0.44) compared to those without any symptoms or a mild course (Online Resource Table 2).

## Discussion

We observed that PCC recovery was faster for the newer virus variants, while vaccinations preceding infection were not independently associated with recovery. Respondents infected during Omicron dominance reported a faster recovery than those infected earlier in the pandemic. This result is in accordance with research describing a lower PCC risk for the Omicron strain [3, 6, 8, 11–13, 16], the results of Morello et al. (2023) who observed a faster PCC recovery in children infected with Omicron [22], and the results of Atchison et al. (2023), who observed a prolonged recovery for those infected with the SARS-CoV-2 wildtype [3].

As expected, vaccination status and virus variant were highly correlated (Online Resource Table 4). Initially, vaccination status appeared to be associated with a faster PCC recovery, but this association disappeared when considering virus variants. This finding is consistent with that of Atchison et al. (2023), who observed a faster recovery among those who received at least two vaccine doses in the crude analysis; however, after adjustment for various factors (e.g., age, sex, comorbidities, virus variant), this effect was no longer evident [3]. Since they adjusted for several factors simultaneously, it was not clear which variable confounded the initially observed association of vaccination and recovery. Our approach allowed us to confirm our assumption that recovery is dependent on the virus variant and probably not on vaccination status.

In some studies on PCC risk, a similar pattern emerged [6, 7, 9, 13, 15]. For example, Reme et al. (2023) reported that vaccination status was not significantly associated with the development of PCC when the virus variant was considered [13]. However, vaccination reduces the risk of severe acute COVID-19 disease [24] which in turn was associated with PCC recovery in our population and with PCC risk in other research [6–10, 12, 16, 17]. Accordingly, vaccination status may influence both PCC risk and recovery rate via this indirect pathway [3, 12].

It is important to note that each virus variant marks a different phase of the pandemic. Ealier infections are probably associated with a higher psychosocial burden, potentially affecting the post-acute course.

About 37% of our population recovered within one year, which is consistent with the 31% reported by Atchison et al. (2023) [3]. Another 6% recovered within the following year, which aligns with the observation that after the first year, the chance of recovery diminishes and PCC essentially becomes a chronic condition [25].

Apart from virus variant and the course of the acute infection, we observed a sex and age effect. Our results are in line with previous research on PCC risk indicating that both women and older individuals have a higher PCC risk than men and younger people [9, 15–17], and with research indicating faster PCC recovery in men [3, 26, 27]. Hartung et al. (2024) reported that being male, older, and less educated were predictors of persistent cognitive deficits in individuals with PCC, suggesting symptom-specific predictors of recovery and highlighting the importance of further research [28].

The majority of our cohort is expected to have mild to moderate PCC. Participation in a survey requires cognitive effort and can be tiring. This could lead to a lower participation of those with severe fatigue. At the same time, survey participation requires motivation, which may be higher in a more affected population. These aspects should be considered when interpreting our results.

### Strengths and limitations

While previous studies focused primarily on risk factors for PCC, we were able to extend this by considering risk factors for persistent symptomatology in people with PCC. We used data from a large cohort study and were able to gain insights over a long observation period. By focusing on the virus variant, we emphasized the importance of considering virus variants when studying PCC recovery.

It should be noted that all our data are based on self-reports, not on confirmed SARS-CoV-2 infections or PCC diagnoses. Although such self-reports do not provide objective assessments, they offer valuable insights into individual experiences. This aspect is particularly relevant for PCC, as it is still a relatively poorly understood condition associated with substantial limitations in everyday life [3–5].

We did not consider fourth vaccine doses separately. However, a protective effect of a fourth dose has been reported in studies on PCC risk that considered both the virus variant and specifically a fourth vaccine dose [11, 12].

We did not assess comorbidities. However, these appear to be an important predictor of both PCC risk and recovery [3, 9, 10, 13, 14, 17, 26, 27]. As people with comorbidities have a higher risk of a more severe acute course, we may have been able to account for some of this effect by adjusting for this variable. Nevertheless, adjusting for comorbidities did not eliminate the association between variant and PCC recovery in another study [3].

We considered people “recovered” when they reported no ongoing symptoms. This approach does not consider the possibility of symptoms subsiding and (re-)appearing, posing the potential for misclassification as “recovered”. However, we tried to counter this bias by asking about symptomatology in the past four weeks at the follow-up (Online Resource Table 5). Furthermore, the most frequently observed course of PCC is that symptoms slowly improve over time [29].

### Conclusion

We observed a faster PCC recovery after infection with Omicron compared to earlier variants. After accounting for virus variants, we did not find an independent effect of vaccination status on recovery. Less than 40% of respondents were recovered one year post-infection. This indicates a high proportion of cases with chronification of symptoms, highlighting the importance of ongoing research regarding PCC recovery.

## Electronic supplementary material

Below is the link to the electronic supplementary material.

Supplementary Material 1

## Data Availability

The data underlying this work are not publicly available due to data sensitivity. However, the data can be obtained from the corresponding author upon reasonable request.
