# Supplementary material for: Post-COVID recovery is faster after an infection with the SARS-CoV-2 Omicron variant: a population-based cohort study
Source: Infection. 2024 Nov 18;53(2):657–65. doi: 10.1007/s15010-024-02438-z (PMC11971134; doi:10.1007/s15010-024-02438-z)
Supplement: Supplementary file 1 — Supplementary Material 1 [file 15010_2024_2438_MOESM1_ESM.pdf]

Supplementary Information to

Post-COVID Recovery is Faster After An Infection With The SARS-CoV-2 Omicron Variant: A Population-Based Cohort Study

Laura R. Pfrommer, MSc; Sophie Diexer, MSc; Bianca Klee, MPH; Janka Massag, MSc; Dr. Cornelia Gottschick; Dr. Oliver Purschke; Prof. Dr. med. Mascha Binder; Prof. Dr. med. Thomas Frese; Prof. Dr. med. Matthias Girndt; Prof. Dr. med. Daniel Sedding; Prof. Dr. med. Jonas Rosendahl; PD Dr. med. Jessica I. Hoell; Dr. Irene Moor; Prof. Dr. med. Michael Gekle; Dr. med. Christine Allwang; Prof. Dr. med. Florian Junne; Prof. Dr. med. Rafael Mikolajczyk*

Corresponding Author:
Rafael Mikolajczyk
Institute for Medical Epidemiology, Biometry and Informatics (IMEBI)
University Medical Center, Martin Luther University Halle-Wittenberg
Magdeburger Str. 8 in 06112 Halle (Saale), Germany
Phone: +49 (345) 557-3571
E-mail: rafael.mikolajczyk@uk-halle.de

Table of contents

Table 1 Potential post-acute COVID-19 condition symptoms at ≥12 weeks post-infection available for selection **2**

Table 2 Results of the first sensitivity analysis (Cox regression for interval-censored data; numbers above 1 indicate a faster recovery) **3**

Table 3 Results of the second sensitivity analysis (Cox regression for interval-censored data; numbers above 1 indicate a faster recovery) **4**

Table 4 Contingency table of COVID-19 vaccination status prior to infection and dominant SARS-CoV-2 variant at infection in the DigiHero post-acute COVID-19 cohort included in analyses (N = 4,529) **5**

Table 5 Potential ongoing post-acute COVID-19 condition symptoms in the last four weeks available for selection (presented at the follow-up) **6**

References **7**

Table 1 Potential post-acute COVID-19 condition symptoms at ≥12 weeks post-infection available for selection

	not at all	very mild	mild	moderate	severe	do not know
Fever						
Swelling of lymph nodes						
Smell and taste disorders						
Joint, muscle or limb pain						
Tiredness, fatigue (with a normal amount of sleep)						
Sleep disorder						
Night sweats						
Cognitive impairments (concentration difficulties, memory loss, confusion)						
Anxiety						
Depression						
Headache						
Common cold						
Conjunctivitis						
Earache or ringing in the ears (tinnitus)						
Shortness of breath						
Sore throat						
Cough						
Tightness in the chest/ chest pain						
Heart problems such as palpitations or arrhythmia						
Vertigo						
Stomach ache						
Gastrointestinal complaints/ diarrhea						
Nausea						
Premenstrual syndrome (PMS)/ menstrual cramps						

Note. The list was presented to DigiHero respondents at the infections-assessment (roll-out: August, 2021).

Table 2 Results of the first sensitivity analysis (Cox regression for interval-censored data; numbers above 1 indicate a faster recovery)

A. Considering vaccination status, not variant (N=2,275)	crude HR	95%CI		adjusted HR[a]	95%CI	
Vaccination status prior to infection (ref: not vaccinated, n=1,176)						
Received one dose (n=80)	1.10	0.75	1.63	1.06	0.72	1.55
Received two doses (n=417)	1.54	1.26	1.88	1.46	1.19	1.79
Received booster dose (n=602)	1.75	1.43	2.15	1.57	1.27	1.94
**B. Analyses stratified for virus variant**	crude HR	95%CI		adjusted HR[a]	95%CI	
**B.1 Omicron variant only (N=796)**						
Vaccination status prior to infection (ref: not vaccinated, n=52)						
Received one dose (n=21)	0.64	0.09	4.61	0.62	0.08	4.59
Received two doses (n=141)	1.17	0.72	1.90	1.12	0.66	1.90
Received booster dose (n=582)	0.92	0.60	1.41	0.88	0.57	1.37
**B.2 Delta variant only (N=434)**						
Vaccination status prior to infection (ref: not vaccinated, n=127)						
Received one dose (n=20)	0.99	0.43	2.29	0.65	0.26	1.65
Received two doses (n=267)	0.77	0.53	1.11	0.62	0.41	0.96
Received booster dose (n=20)	0.68	0.26	1.79	0.41	0.15	1.13
**B.3 Alpha variant only (N=508)**						
Vaccination status prior to infection (ref: not vaccinated, n=460)						
Received at least one dose (n=48)	0.86	0.46	1.60	0.92	0.45	1.87
**B.4 SARS-CoV-2 wildtype or Alpha variant (N=1,045)**						
Vaccination status prior to infection (ref: not vaccinated, n=997)						
Received at least one dose (n=48)	0.83	0.42	1.67	0.94	0.46	1.91
**C. Considering variant, not vaccination status (N=2,275)**	crude HR	95%CI		adjusted HR	95%CI	
**Dominant variant at infection** (ref: SARS-Cov-2 wildtype or Alpha, n=1,045)						
Delta (n=434)	1.67	1.35	2.06	1.68	1.36	2.07
Omicron (n=796)	2.32	1.85	2.90	2.13	1.71	2.65
**Sex** (ref: male; n=582)						
Female (n=1,693)				0.79	0.67	0.94
**Age** (ref: <30; n=248)						
30-39 (n=385)				0.86	0.63	1.18
40-49 (n=498)				0.64	0.46	0.87
50-59 (n=696)				0.70	0.51	0.95
60-69 (n=354)				0.72	0.51	1.02
≥70 (n=94)				0.75	0.49	1.15
**Education level** (ref: high; n=1,293)[b]						
Low (n=80)				0.96	0.58	1.59
Medium (n=853)				0.91	0.79	1.06
Not available (n=49)				0.65	0.31	1.36
**Net household income in €** (ref: <2.250; n=472)						
2.250 to <4.000 (n=900)				1.06	0.84	1.34
≥4.000 (n=711)				1.19	0.94	1.51
Not available (n=192)				1.09	0.78	1.53
**Course of acute disease** (ref: no symptoms/mild course; n=746)						
moderate course (n=1,144)				0.68	0.59	0.79
severe course (n=385)				0.33	0.24	0.44

Note. The first sensitivity analysis considered DigiHero respondents with post-acute COVID-19 condition at the infections-assessment who provided complete information on the acute course of their COVID-19 disease (N = 2,275). Hazard ratios (HR) and 95% confidence intervals (95%CI) are shown.

[a] Analyses adjusted for age, sex, educational level, household income, and course of acute COVID-19 disease.

[b] The education level was defined based on the International Standard Classification of Education (ISCED-97) [1]

Table 3 Results of the second sensitivity analysis (Cox regression for interval-censored data; numbers above 1 indicate a faster recovery)

**A. Considering vaccination status, not variant (N=2,177)**	crude HR	95%CI		adjusted HR[a]	95%CI	
Vaccination status prior to infection (ref: not vaccinated, n=745)						
Received one dose (n=67)	0.84	0.41	1.72	0.85	0.41	1.77
Received two doses (n=310)	1.42	1.05	1.92	1.39	1.03	1.88
Received booster dose (n=1,055)	2.21	1.80	2.72	2.13	1.72	2.64
**B. Analyses stratified for virus variant**	crude HR	95%CI		adjusted HR[b]	95%CI	
**B.1 Omicron variant only (N=1,292)**						
Vaccination status prior to infection (ref: not vaccinated, n=51)						
Received one dose (n=28)	0.34	0.00	91.96	0.28	0.00	77.67
Received two doses (n=166)	0.82	0.46	1.48	0.68	0.38	1.25
Received booster dose (n=1,047)	1.08	0.66	1.80	0.95	0.56	1.59
**B.2 Delta variant only (N=247)**						
Vaccination status prior to infection (ref: not vaccinated, n=79)						
Received at least one dose (n=168)	0.69	0.39	1.22	0.62	0.34	1.14
**B.3 Alpha variant only (N=313)**						
Vaccination status prior to infection (ref: not vaccinated, n=290)						
Received at least one dose (n=23)	0.51	0.00	672.87	0.54	0.00	819.68
**B.4 SARS-CoV-2 wildtype or Alpha variant (N=638)**						
Vaccination status prior to infection (ref: not vaccinated, n=615)						
Received at least one dose (n=23)	0.50	0.00	962.84	0.49	0.00	1055.44
**C. Considering variant, not vaccination status (N=2,177)**	crude HR	95%CI		adjusted HR	95%CI	
**Dominant variant at infection** (ref: SARS-Cov-2 wildtype or Alpha n=638)						
Delta (n=247)	1.68	1.18	2.38	1.81	1.27	2.58
Omicron (n=1,292)	2.61	2.04	3.32	2.57	2.00	3.30
**Sex** (ref: male; n=514)						
Female (n=1,663)				0.91	0.76	1.11
**Age** (ref: <30; n=276)						
30-39 (n=363)				0.75	0.56	1.01
40-49 (n=456)				0.42	0.31	0.58
50-59 (n=634)				0.51	0.38	0.67
60-69 (n=338)				0.59	0.42	0.84
≥70 (n=110)				0.52	0.33	0.83
**Education level** (ref: high; n=1,135)[c]						
Low (n=82)				1.06	0.68	1.65
Medium (n=915)				0.96	0.82	1.12
Not available (n=45)				0.84	0.43	1.63
**Net household income in €** (ref: <2.250; n=487)						
2.250 to <4.000 (n=804)				1.16	0.93	1.45
≥4.000 (n=683)				1.58	1.25	1.99
Not available (n=203)				1.07	0.76	1.50

Note. The second sensitivity analysis considered DigiHero respondents with post-acute COVID-19 condition at the infections-assessment who indicated experincing at least one post-acute COVID-19 symptom they rated as "severe" (N = 2,177). Hazard ratios (HR) and 95% confidence intervals (95%CI) are shown.

[a] Analyses adjusted for age, sex, educational level, and household income

[b] Analyses adjusted for age, sex, and household income

[c] The education level was defined based on the International Standard Classification of Education (ISCED-97) [1]

Table 4 Contingency table of COVID-19 vaccination status prior to infection and dominant SARS-CoV-2 variant at infection in the DigiHero post-acute COVID-19 cohort included in analyses (N = 4,529)

**COVID-19 vaccination status prior to infection**	**Dominant SARS-CoV-2 variant at time of infection**							
	SARS-CoV-2 wildtype		Alpha		Delta		Omicron	
Not vaccinated	709	100%	595	91.4%	175	28.9%	114	4.4%
One dose	-	-	45	6.9%	43	7.1%	48	1.9%
Two doses	-	-	11	1.7%	359	59.3%	322	12.6%
Three or more doses	-	-	0	-	28	4.6%	2,080	81.1%
**Total**	709	100%	651	100%	605	100%	2,564	100%

Note. The correlation between the two variables is r = 0.86 (Spearman correlation).

Table 5 Potential ongoing post-acute COVID-19 condition symptoms in the last four weeks available for selection (presented at the follow-up)

	complaint not present	not impaired	mildly impaired	strongly impaired
Stomach ache				
Back pain				
Pain in the arms, legs, or joints (knees, hips, etc.)				
Menstrual pain or PMS (premenstrual syndrome)				
Pain or problems during sexual intercourse				
Headache				
Chest pain				
Vertigo				
Fainting spells				
Palpitations or tachycardia				
Shortness of breath				
Constipation, nervous bowel, or diarrhea				
Nausea, flatulence, or digestive problems				
Difficulty falling asleep or staying asleep, or increased sleep				
Fatigue or the feeling of not having energy				
Rapid exhaustion				
Concentration difficulties				
Memory impairments				
Reduced physical performance				
Hypersensitivity to noise or light				
Circulatory problems such as dizziness, weakness, or palpitations when standing up				
Sensory disorders such as tingling sensations, burning, or numbness				
Muscle twitching				
Muscle weakness				

Note. The list was presented to DigiHero respondents at the follow-up assessment (roll-out: December, 2022).

References

1 Organisation for Economic Co-Operation and Development. Classifying Educational Programmes: Manual for ISCED-97 Implementation in OECD Countries. 1999 Edition.
